# Benefits of a joint health sciences practicum for students in library and information sciences: a case report

**DOI:** 10.5195/jmla.2020.720

**Published:** 2020-01-01

**Authors:** Rebecca Raszewski, Jonna Peterson

**Affiliations:** Associate Professor and Information Services & Liaison Librarian for Nursing, Library of the Health Sciences Chicago, University of Illinois at Chicago, Chicago, IL, raszewr1@uic.edu; Senior Clinical Informationist, Galter Health Sciences Library and Learning Center, Feinberg School of Medicine, Northwestern University, Chicago, IL, jonna.peterson@northwestern.edu

## Abstract

**Background:**

A joint practicum gives library and information science (LIS) students the opportunity to compare two health sciences libraries’ structures and workflows. The goal of this case report is to describe how a joint health sciences practicum can help LIS students and recent graduates develop skills that may be beneficial for their future positions in health sciences or other libraries.

**Case Presentation:** Six participants in a joint health sciences library practicum underwent two interviews: the first interview focused on their practicum experiences, and the second interview sought to determine whether the participants had found employment and were using any skills in their new positions that they acquired during their practicums. Participants gave mostly positive feedback regarding their practicum experiences and expressed openness to applying for health sciences library positions. Although the participants who found employment did not work in health sciences libraries, their practicum projects served as supporting materials for their job applications, and they were using the skills they had gained from their practicums in their new positions.

**Conclusions:**

While most joint practicum participants were not working in a health sciences library, the practicum was beneficial to their new careers. This case report highlights that a joint health sciences practicum program can be beneficial in showing LIS students different approaches to health sciences librarianship.

## BACKGROUND

A joint practicum is a structured fieldwork experience that allows library and information science (LIS) students to compare two libraries by observing their organizational structures and workflows and to network with librarians from different departments. These practicums require the discipline and flexibility of both the LIS students and practicum supervisors in completing the practicum at both locations in a fixed amount of time. When the student receives academic credit, site supervisors are required to complete and submit paperwork on the student’s behalf and to host the student’s academic advisor for a site visit, when required.

Joint practicums have received little attention in the LIS literature. Although articles on joint practicums have been written from the student’s perspective, they have not contained any analysis [1, 2]. Previous studies examining the practicum experiences of LIS students or recent graduates have been conducted in health sciences libraries to obtain thoughts on their fieldwork experiences, suggestions for improvement, or perceptions of the fieldworks’ impact on their future careers [3–8]. The joint practicum at Rush University Library and University of Illinois at Chicago (UIC) Library of the Health Sciences Chicago (LHSC) is the sole example of a joint health sciences practicum in the LIS literature [9].

Joint health sciences library practicums can give LIS students a comprehensive view of health sciences librarianship that they might not experience through other aspects of their educational programs. Forty-six of sixty American Library Association–accredited LIS programs include health sciences librarianship education—most frequently stand-alone courses—whereas fourteen do not offer anything in health sciences librarianship [10]. In a 2015 forum on re-visioning LIS education, Abels, Howorth, and Smith specified increasing opportunities in fieldwork and providing new forms of these educational experiences as an action item [11]. Thus, a joint practicum in health sciences librarianship could be a viable action item for LIS education.

The goal of this case report is to describe the impact of Rush University Library and LHSC’s joint health sciences practicum experiences on participants’ professional skills and whether they ended up working in health sciences libraries.

## CASE PRESENTATION

The joint health sciences practicum at Rush University Library and LHSC that began in 2011 gave LIS students the opportunity to compare a private, single-site university with a large, public multicampus university (Table 1).

**Table 1:** Practicum site comparison

Two health sciences libraries	Rush University Library	University of Illinois at Chicago (UIC) Library of the Health Sciences Chicago (LHSC)
Type of institution	Private	Public
Number of libraries	Single library	Multiple-library system
Location of library	Within hospital	Stand-alone
Number of health sciences colleges	Four colleges: nursing, health sciences, medicine, graduate school	Seven colleges: applied health sciences, dentistry, medicine, nursing, public health, pharmacy, social work
Librarian status	Staff	Faculty
Number of hospital beds	884	495
Number of library staff	Librarians: 8 Nonlibrarian staff: 19	Librarians: 16 Nonlibrarian staff: 16

Eight participants completed the practicum between October 2011 and April 2015. Six were LIS students, and 2 were recent graduates of LIS programs. Five of the 6 LIS students received academic credit for completing the practicum. Typically, participants began their practicums at Rush University, the smaller of the 2 institutions. The length of the practicum, which ranged from 100 to 120 hours, depended on the LIS program’s requirements and was split between institutions. Practicum participants did not need formal training in health sciences resources, only an interest in learning more about health sciences librarianship.

The practicum focused on the public services side of health sciences librarianship at each institution. Participants were introduced to health sciences databases, citation management tools, and point-of-care tools that were unique to each institution through either one-on-one training or observing research consultations and workshops. They also participated in in-person and virtual reference transactions and attended library events and morning report in the department of internal medicine. Practicum supervisors arranged meetings with librarians and staff in other departments, such as circulation or special collections, so that participants could network and learn more about these departments. Participants were assigned projects that included creating subject guides using LibGuides, creating instructional materials, or designing classes on health sciences resources. Participants were responsible for detailing their experiences and observations in a weekly report and a cumulative presentation for librarians and staff at both locations.

After participants completed their final practicum presentations, they were offered the opportunity to participate in a follow-up study consisting of two fifteen-to-twenty-minute phone interviews, for which they provided informed consent as approved by each institution’s institutional review board office. Interviews were conducted between December 2012 and May 2016. The first interview occurred at the end of their practicums or after they graduated from their LIS programs, and the second interview occurred approximately one year later. To minimize bias, interviews were conducted by a faculty or staff member at UIC who was not involved in the practicum. Interview questions are provided in the supplemental appendix. The interviewer took notes on participants’ responses and entered the responses into a spreadsheet. Each participant was randomly assigned a letter for reporting their responses in this article. Two of the eight participants contacted for interviews never responded. Five of these six respondents completed the first interview. Although all six participants completed the second interview, some interview data were lost, resulting in four second interviews for analysis.

Participants’ responses to interview questions were grouped under the following thematic categories: joint practicum experience, acquired skills and challenges, participants’ projects, impressions of health sciences librarianship, and one year later.

### Joint practicum experience

Most participants said having their practicums at two locations enhanced their experience. Participant E felt that her experience was enhanced because she “valued being able to meet so many librarians.” She also developed “a clearer picture of what I’ll do better in the future.” Participant E could see the differences between libraries due to their size, with one library having publication and scholarship requirements for librarians. Many participants commented on having difficulty understanding the structure of LHSC and its tenure process, although one indicated she was less surprised when she saw the structure at her new workplace. Exposing students to tenure-track and nontenure-track library positions resulted in some interesting comments: “tenure track is insane!,” said one participant.

### Acquired skills and challenges

When asked which new skills the participants had learned during the practicum, four of the five participants in the first interview mentioned acquiring database searching skills or learning about new resources. PubMed was the only resource named more than once. In addition to PubMed, participant G mentioned learning new terminology and handling reference questions over the phone. Participant L answered that the practicum “expanded goals in where I wanted to work in general to include health sciences.”

Participants’ responses varied in what their biggest challenges were during their practicums. While participant L said that the “practicum was not challenging,” participant G said, “all [of it] was a challenge because [I had] never done it before.” She also mentioned the amount of time it took to understand PubMed and “ongoing education on keeping up with updates.” Participant E’s biggest challenge was getting “frustrated that instructions needed reclarifying” for her projects. Participant C, who had created a tutorial using Adobe Captivate for one of her projects, mentioned that learning Adobe Captivate was her biggest challenge. Participant A’s biggest challenge was “taking it all in.” The more specific challenges she listed were “medical terminology, how different departments fit and work together,” and “how tenure works.”

When asked what they were least prepared for during the practicum, participants listed “creating videos” and “sitting at the reference desk.” Participant L was the only one who said she “felt prepared to understand concepts” and that “reference was scary but I was prepared.” Participant G said she felt least prepared to sit at the reference desk. Participant C mentioned “the commute and learning everyone’s name.” Also, while she did not teach during the practicum, participant E shadowed a few librarians and “was not prepared for how much teaching health sciences librarians do.”

### Participants’ projects

When asked if the projects given to participants were helpful, two participants mentioned their use of LibGuides to create subject guides. Participant L said, “including original material is good to put on [my] resume.” Participant E said that writing a script for a video tutorial was a good skill to have because was she doing distance/online teaching in her new position. Participant C answered that learning Adobe Captivate and creating a handout on copyright implications were helpful.

### Impressions of health sciences librarianship

When asked if the practicum changed their views of health sciences librarianship and what their views were after finishing the practicum, participant L said that there was “pretty good respect for its importance.” Her views on health sciences librarianship did not change, but she was more interested in the profession. Participant C said that there was more instruction than she thought there would be and that health sciences librarians were “underutilized but fabulous resources.” Participant A was not “aware” of health sciences libraries before her practicum, but they “seemed more approachable now.” Participant E stated that the practicum opened her eyes to the importance of librarians in academic libraries. She said she expected to really like health sciences librarianship but left her practicum loving it. She commented that she initially thought that health sciences librarianship was more “static” but then saw how “dynamic” it was. She listed morning report and research opportunities as examples of dynamic environments.

Participants were open to working in a health sciences library, which became another option for them during their job searches. At the time of the first interview, participants were applying to health sciences library positions when they were available. Participant L said that after her experience she was open to working in a health sciences library. She indicated she was applying for positions in both public and health sciences libraries but felt that health sciences opportunities required more experience. All participants were interested in applying to health sciences libraries positions but realized the health sciences library job market could be narrow.

### One year later

Although most participants were able to get jobs after graduation, none were working in a health sciences library. One participant was working full-time in a special library. Another participant was working part-time in a public library. One participant had obtained a full-time position in an academic library. One participant had not yet obtained a library position at the time of the second interview but was hired for a part-time position at an institution shortly thereafter.

The projects that were completed during their practicums gave participants materials they could highlight when they applied for positions. One participant used the instructional videos and LibGuides she created to provide content for her application portfolio. Participant P believed that the work she completed during the practicum was instrumental to obtaining a full-time position as a product literature specialist in a special library where she managed a team of six people. The practicum provided her some basics that she used in her new position, including document delivery. Another participant used a Scopus database handout she created for her application for her current position. She appreciated “all the care and effort all the librarians took in me, and that all the products I created would be useful for students. It was a lot of work to take on a practicum student, and it jump-started my career.”

## DISCUSSION

This case report demonstrates that a joint health sciences library practicum experience can be beneficial to LIS students and recent graduates by giving them an opportunity to gain transferable skills and experience for their future careers. Practicum projects are useful for participants to use as supporting materials for their resumes. A few participants commented that the practicum helped them obtain their first librarian position. A joint health sciences practicum gives LIS students and recent graduates an opportunity to compare each library’s organizational structure and similar departments’ workflow. Participants may see different approaches for searching databases such as PubMed or be exposed to more library resources, as each institution may have different databases or point-of-care tools. They can also network with more librarians as they interact with various departments at each institution.

The experiences described in this case report are similar to other reported fieldwork experiences for LIS students. When Gariepy surveyed LIS internship program participants about their current careers, 67% felt their internships had a significant impact on obtaining their first jobs [6]. In Ferrer-Sobel and Vinent’s study of an academic library’s practicum students, almost half of the students felt the practicum was a major factor in successfully securing a professional position [12]. In Keselman et al.’s study of interns and graduates from a Latino- and Native American–focused internship, the majority of the participants “felt that the internship furthered their careers” or “provided them with specific tangible skills and resume-strengthening experience,” with some participants saying that “the internship gave them professional confidence and equipped them with a network and connections for job searches” [8]. In Bastian’s survey of former archival internship students, most agreed that the internship helped them better understand the archival profession, and the internship experience on their resume “made them attractive to employers” [13].

Over the last thirty years, studies have shown that practicums or other fieldwork experiences are not always required in LIS programs [14–18]. In more recent studies, Bird et al. found that apart from school libraries programs, only ten of fifty-nine North American LIS programs required an internship but that LIS programs from countries outside of North American tended to require a practicum [19]. In Huggins’ study of end-of-program assessments in LIS programs, twenty-five of fifty-eight programs reported that internships were a requirement for their students [20].

Therefore, practicums and other fieldwork experiences need to be further emphasized or required by LIS programs, because they help students apply what they learn in LIS programs in workplace settings, thereby filling gaps in their LIS education, which is illustrated by comments from two of our participants about their practicum experiences. One student said, “in health sciences librarianship, you work hand in hand with doctors and students. Before, I didn’t understand the interaction or how involved librarians could be.” Another student thought her practicum experience would be “boring,” but it “was very eye opening as to how much instruction and 1-on-1 interaction [health sciences] librarians have. Also [I am] amazed at how much they work with doctors and faculty finding information.” Practicums and other field work experiences should also be structured and assessed, especially to ensure that LIS students are working on projects that may be beneficial for future work experience.

A joint practicum can be adapted into other forms of LIS fieldwork experiences. LIS students could compare educational opportunities or services for a similar patron group at different institutions, providing feedback or creating projects based on their observations. Despite some examples of virtual internships in LIS education [21–23], they have focused on only a single location. However, educational technology systems can support a joint practicum or other educational experience, especially for LIS students who are located in rural or remote areas. Another possibility is that LIS students could participate in a practicum focusing on virtual reference or distance education support at two institutions. Coltrain’s article on LIS students offering distance education support and Purpur and Morris’ article on LIS students building reference interview skills through virtual reference could be used as models for expanding to a joint practicum experience [24, 25].

The small number of students and recent graduates described in this case report are not reflective of the entire LIS student population. Participants’ responses might have differed if their practicums were conducted at other locations. Our joint practicum is offered twice each year but is not always possible due to external factors such as construction projects, a lack of applicants, or scheduling conflicts for practicum supervisors.

In 2015, one of the authors started a new position at Northwestern University’s Galter Health Sciences Library. Since 2015, five students have participated in the joint practicum at its new homes at Galter Library and LHSC. In conjunction with the supporting LIS programs, the authors would like to explore additional methods of evaluating the joint practicum in the future. Future research could include a thematic analysis of students’ weekly reports. A formal evaluation of the students’ cumulative presentation may also be valuable. Furthermore, the Medical Library Association (MLA) Competencies Self-Assessment tool [26], which introduces participants to the revised *MLA Competencies for Lifelong Learning and Professional Success* [27], could be either used or modified as an evaluation tool for practicum students.

## Supplemental File

Appendix Interview questions for participants

## 

**Rebecca Raszewski, MS, AHIP,** raszewr1@uic.edu, http://orcid.org/0000-0003-1210-4272, Associate Professor and Information Services & Liaison Librarian for Nursing, Library of the Health Sciences Chicago, University of Illinois at Chicago, Chicago, IL

**Jonna Peterson, MLIS,** jonna.peterson@northwestern.edu, https://orcid.org/0000-0001-6585-892X, Senior Clinical Informationist, Galter Health Sciences Library and Learning Center, Feinberg School of Medicine, Northwestern University, Chicago, IL
