# Supplementary material for: Benefits of a joint health sciences practicum for students in library and information sciences: a case report
Source: J Med Libr Assoc. 2020 Jan 1;108(1):106–12. doi: 10.5195/jmla.2020.720 (PMC6920000; doi:10.5195/jmla.2020.720)
Supplement: Appendix [file jmla-108-106-s001.pdf]

JMLA Journal of the Medical Library Association

SC

Supplemental content to
J Med Libr Assoc. Jan;108(1):dx.doi.org/10.5195/jmla.2020.720
www.jmla.mlanet.org
© Raszewski, Peterson 2020

Benefits of a joint health sciences practicum for students in library and information sciences: a case report

Rebecca Raszewski, MS, AHIP; Jonna Peterson, MLIS

APPENDIX

Interview questions for participants

Interview questions to be asked at end of practicum/graduation

1. During your practicum, what new skills or interests did you discover?
2. What were your biggest challenges?
3. What were you least prepared for in the practicum?
4. How were the projects you were given helpful?
5. Did the experience of doing the practicum in two libraries detract from or enhance your experience? Why or why not?
6. What are your views of health sciences librarianship now after you have completed the practicum?
7. How has the practicum changed your views of health sciences librarianship?
8. Will you apply for health sciences librarian positions when you graduate? Why or why not?
9. When do you expect to graduate? When did you graduate?
10. Please share any additional thoughts about the practicum.

Interview questions to be asked at one year from the end of practicum/graduation

1. Are you currently employed in a library? If so, where?
    a. If students do not have a position:
        i. If you are not currently employed in a library, have you been applying to health sciences libraries?
        ii. What kind of position are you seeking?
2. Is your status full- or part-time?
3. What is your title?
4. Please describe your major job responsibilities.
5. What skills did you learn during the practicum that you are using at your current position?
6. What skills did you wish the practicum would have covered that would have helped you during your current position?
7. Are you considering serving as a mentor for a practicum student?
8. Is your workplace amenable to providing a practicum experience for a student?
